# Recruitment and retention of young adult veteran drinkers using Facebook

**DOI:** 10.1371/journal.pone.0172972

**Published:** 2017-03-01

**Authors:** Eric R. Pedersen, Diana Naranjo, Grant N. Marshall

**Affiliations:** RAND Corporation, Department of Behavioral and Policy Sciences, Santa Monica, CA, United States of America; California Pacific Medical Center Research Institute, UNITED STATES

## Abstract

The objective of this study was to describe the feasibility of using Facebook as a platform to recruit and retain young adult veteran drinkers into an online-alcohol use intervention study. Facebook’s wide accessibility and popularity among the age group that comprises the majority of veterans from the conflicts in Iraq and Afghanistan make it a compelling resource through which research can extend its reach to this otherwise hard-to-reach group. We developed a series of Facebook advertisement campaigns to reach veteran drinkers not specifically searching for alcohol treatment. In doing so, we recruited 793 valid veteran participants in approximately two weeks for an advertising cost of $4.53 per obtained participant. The study sample consisted primarily of male veterans, between 19 and 34 years of age, who were drinking at moderate to heavy levels. Although about half of the sample reported mental health comorbidity, few had received any mental health or substance use treatment in the past year. Facebook appears to be a valuable mechanism through which to recruit young veterans with unmet behavioral health needs, although more specific efforts may be needed to engage certain types of veterans after initial study enrollment.

## Introduction

American veterans, specifically those who served most recently in Operation Enduring Freedom and Operation Iraqi Freedom (OEF/OIF), are vulnerable to myriad behavioral health disorders. Substance use disorders (SUDs), posttraumatic stress disorder (PTSD), and depressive disorders are among the most common diagnoses found among these veterans enrolled in the Veterans Affairs Health Care System (VHA) [1], and those meeting criteria for one of these behavioral health problems are at increased risk for meeting criteria for others [2–4]. These problems affect the young veteran population at disproportionate rates when compared to their peers in active duty and older veterans [5–9].

Connecting young veterans to care for these behavioral health disorders is of paramount importance as proper care can provide much needed relief during reintegration post-discharge. However, a large proportion of OEF/OIF veterans in the United States do not seek care. For example, data from the VHA reveals that about 39% of all OEF/OIF veterans have never sought VHA care for any reason since separation, and only 37% had used any VHA services during a one year period between 2014 and 2015 [10, 11]. Rates of behavioral health care receipt are particularly troubling and studies indicate that rates of treatment seeking (both at the VHA and with outside providers) among OEF/OIF veterans with documented behavioral health need (i.e., positive screens for SUDs, PTSD, or depression) vary between 39% and 50% [9, 12–14]. Younger veterans are also less likely than older veterans to seek behavioral health care at the VHA or elsewhere [15, 16]. Thus, finding ways to reach young veterans for behavioral health outreach and research efforts is essential to provide comprehensive and far-reaching services to current and future veterans.

### Study recruitment using Facebook

Nearly all veterans in both VHA and non-VHA samples report access to and use of the Internet, with the majority using the Internet daily and over two-thirds reporting routine use for receiving health information or finding services [17–19]. The website Facebook remains a widely used social media networking site among young adult Internet users, with 89% of young adult Internet users reporting use of the site [20]. Daily activity on the site is high worldwide, with 1.09 billion daily users on average; monthly activity is even higher, especially among mobile monthly active users (1.51 billion) [21]. The population of Facebook users is heterogeneous, including similar rates of use within each group among Whites, African-Americans/Blacks, and Hispanic/Latino(a)s.

Researchers have begun using Facebook for recruitment and program delivery, with studies successfully targeting difficult-to-reach populations such as sexual minorities [22], immigrant groups [23], and youth affected by violence [24]. The site has also been used for recruitment of participants into behavioral health intervention studies, such as smoking cessation programs [25], online programs for depressed individuals [26], programs to promote condom use and deliver HIV prevention messages [27–29], and alcohol reduction programs with college students [30].

Facebook’s wide accessibility and popularity among the age group that comprises the majority of OEF/OIF veterans make it a compelling resource through which research can extend its reach to this otherwise hard-to-reach group. Though no population-level data exist on Facebook use among young veterans, groups on Facebook such as veteran service organizations like Iraq and Afghanistan Veterans of America, have over half a million followers (over 536,000 as of November 2016). In addition, pages targeted toward military family and friends are popular (e.g., Military Spouse Central has nearly 126,000 followers as of November 2016). Facebook reported that in 2014 about 4 million of its users in the United States indicated that they were veterans or active duty military, while 12.5 million users indicated they were family members of the population [31]. Thus, a large number of veterans are on Facebook and, perhaps for those that do not have Facebook accounts, their family members are on and can alert them to studies and outreach efforts targeted towards veterans.

Prior work has used Facebook to attract young veterans into research projects. For example, in our own work we recruited 1,023 young adult veterans in about three weeks into an online one-time survey study [32]. Although eligibility criteria were broad (i.e., participants needed to be between 18 and 34 and needed to be United States veterans separated from the Air Force, Army, Marine Corps, or Navy), approximately 70% screened positive for possible PTSD, depression, or hazardous alcohol use [32, 33]. The study suggested that Facebook can be used to reach veterans with behavioral health concerns outside of traditional recruitment settings and veterans that are not receiving care within the VHA. In our study, only about one-half of the veteran participants screening positive for mental health concerns (PTSD, depression, anxiety) reported receiving any mental health in the past year, while about one-fifth screening positive for hazardous drinking reported past year substance use care. Others have used Facebook to recruit veterans interested in online approaches to reduce alcohol misuse and symptoms of PTSD [34]. For this effort, researchers were able to enroll 600 veterans in just 46 days using targeted Facebook advertising (mean age of approximately 32). The majority of these participants (62%) reported use of behavioral health services in the three months prior to the intervention. These services included inpatient or residential treatment, or outpatient counseling as well as medication and/or self-help groups for mental health concerns and/or substance use. Thus, as Facebook ads contained language about receiving help for alcohol use and PTSD, this study appeared to attract either those looking for adjunct treatments to what they were already receiving or stand-alone treatment outside of traditional in-person settings. Yet many young veterans report barriers to seeking care (e.g., concerns about cost, peers or family members would look down on them if they sought care) [9, 13, 35–41] and Facebook has yet to be studied as a mechanism to reach those not specifically looking for help. Research is also needed to learn how Facebook can be used to recruit an in-need sample that can be retained for follow-up, as our prior work included a brief, one-survey of a general sample of young veterans.

### The present study

The objective of this study was to describe the feasibility of using Facebook as a platform to recruit and retain young adult veteran drinkers into an online-alcohol intervention study. The intervention was designed to attract heavy drinking veterans that were not necessarily looking for treatment [42, 43]. We expand on the initial study findings from our prior work [32] and that of others [34], which demonstrated promising results with regard to the feasibility of recruiting young veterans via social media for survey and intervention research work. This study builds on previous efforts by exploring three key topics related to reaching young veterans in the community for intervention efforts. First, we discuss the advertisement strategy for the Facebook campaign, including efforts to target heavy drinking veterans not specifically searching for care. In the process, we describe the recruitment and retention rates over one month of follow up, as well as the cost and timeliness of recruitment. Given the risk of non-completion during online surveys (i.e., participants begin a survey but drop off prior to completion), we examine factors associated with attrition prior to completing the baseline survey. Second, we describe participant alcohol use behavior, symptoms of behavioral health problems (i.e., mental health problems such as PTSD, substance use problems such as hazardous alcohol use), and use of services to address these behavioral health problems. In doing so, we explore whether Facebook can be used to attract those in need of services that are not receiving them. Lastly, to better understand how best to reach and retain young veterans for research and programmatic efforts, we examine factors associated with dropout from the longitudinal study one-month after initial survey completion.

## Materials and methods

### Participant eligibility criteria

Participants were enrolled for an online study to pilot test a very brief personalized normative feedback intervention (PNF) for young adult veteran drinkers [42, 43]. In this larger study, participants were randomized to receive the PNF or to receive an attention control feedback condition. Treatment effects were evaluated at one month post-intervention. To be eligible for the study, participants needed to be (1) a United States veteran separated from the Air Force, Army, Marine Corps, or Navy, (2) between the ages of 18 and 34, and (3) have access to a computer/mobile phone, Internet, and personal email address. In order to reach veteran drinkers who could benefit from an intervention, participants also needed (4) a score on the 10-item Alcohol Use Disorders Identification Test [44] of 3 for women or 4 for men [45, 46]. AUDIT criteria values were specified to include participants who drank at both moderate to high levels that were at-risk for hazardous or problem drinking. Participants could not currently be affiliated with the military through active duty service or through service in the reserve components of the United States Armed Forces (i.e., Army Reserve or National Guard, Air Force Reserve or Air National Guard, Marine Corps Reserve, Navy Reserve). Those on active duty Coast Guard or Coast Guard Reserve were also ineligible, as were Coast Guard veterans that had not served in one of the other branches of the military (Air Force, Army, Marine Corps, Navy).

### Advertisement and study flow procedures

This study was approved by the RAND Human Subjects Protection Committee (2013–0663). We complied with the terms of service for Facebook for advertising and hosted the online surveys off Facebook on a secure website hosted at the institution. We named the study “Veteran Behaviors Feedback Study” and did not reference treatment for alcohol use in any advertisements to avoid deterring treatment resistant veterans from clicking on ads. The informational page and consent form referred to the intervention only obliquely: “You will also see feedback on-screen after you submit your first survey.”

A series of three Facebook ad campaigns were run to recruit participants into the study. All ads were direct promotions of the survey website, such that an individual who clicked on a Facebook ad would be immediately directed to an external website containing an information page about the study including eligibility criteria, content of the survey questions, and contact information for the Principal Investigator. We also maintained a Facebook page where participants could review this material. This page was reachable from the ads themselves and from a link on the information page. From the information page, participants could click to another page that displayed the consent form. If they consented, then they began a screening survey. If they met eligibility criteria and passed through verification questions (see below), they began the baseline survey. As specified on the information page and in the consent form, only participants who submitted their baseline survey and who met eligibility criteria received the incentive, which was a $20 Amazon gift card delivered via email. Participants who completed a follow-up survey would receive an additional $25 Amazon gift card, for a total of $45. The ad featured the total incentive for study completion.

#### Ads targeting the general population of young veterans

Ads with five different pictures (i.e., helicopters [2 ads], service members walking through a desert, service members skydiving, and a dog tag with the name of the study engraved on it) targeted the general young adult population of veterans. Text of each these ads read, “U.S. veterans aged 18 to 34! Earn $45 for a confidential online study about alcohol use.” Ads were shown to men and women aged 18–40 (age range was larger than 34 to reach family members/spouses) in the United States who used Facebook in English. Keywords to target those interested in or who had “liked” specific content related to American veterans were specified as veteran service organizations (e.g., National Resource Directory, Student Veterans of America, Iraq and Afghanistan Veterans of America) and military spouse pages (e.g., Military Spouse Central).

#### Ads targeting female veterans

To reach female veterans specifically, we launched an ad campaign featuring a picture of hands on a keyboard that stated, “Female veterans! Young adult veterans can receive $45 for completing an online survey research study.” Ads were shown to women between the ages of 18 and 40 in the United States who used Facebook in English. Keywords were specified as those in the veteran service organizations and military spouse pages described above, as well as female-specific Facebook groups such as American Women Veterans and Women in the Military.

#### Ads targeting Air Force and Navy veterans

In our initial efforts using Facebook recruitment for this population, we collected a sample that was over-representative of Army and Marine Corps veterans and under-represented of Air Force and Navy veterans [32]. To enroll more Air Force and Navy veterans, we ran an ad set with two different pictures (i.e., hands on a keyboard, service members walking through a desert) that stated, “Air Force and Navy veterans! Earn $45 for a brief, confidential online survey study.” Ads were shown to men and women between the ages of 18 and 40 in the United States who used Facebook in English. Keywords were specified as those in the veteran service organizations and military spouse pages described above, as well as keywords of United States Air Force, United Stated Navy, and United States Navy Seals.

### Verification of veteran participants

Since there is no face-to-face contact with participants, a concern with Internet-based research is that it is difficult to determine if participants are misrepresenting themselves as meeting eligibility in order to receive an offered incentive. To address this, we developed a series of checks to ensure, to the extent possible, that participants were actual veterans. First, ads were targeted such that they were only displayed to Facebook users who expressed an interest in or “liked” specific Facebook content that was related to American veterans. Second, participants completed three items about their military service that needed to be consistent to progress to the baseline survey, provided that they also met eligibility criteria. Items regarding rank at discharge (e.g., specialist), branch of service (e.g., Army), and pay grade at discharge (e.g., E-4) needed to match. If answers were inconsistent, then they were exited from the survey and could not return to it using the same Facebook account. Participants that failed to meet any of the eligibility criteria (e.g., participant endorsed they were on active duty or in the Coast Guard) were also exited from the survey. Participants could only access the survey once per each Facebook account. In addition, after baseline data were collected, we reviewed the data to make sure that responses to an open-ended item about occupation in the military (i.e., military occupational specialty, enlisted classification, or specialty code), matched the rank, branch, and pay grade items. We verified that rank, branch, pay grade, and occupation matched up using sources from the Department of Veterans Affairs and the Department of Defense websites. We reviewed timestamps on survey start and end times and removed participants that completed the baseline survey in less than eight minutes. Such a timeframe was determined to be impossible given the length of the survey in preliminary tests with research assistants. Lastly, participants completing the follow-up survey one month later responded to items regarding gender, branch, and rank at discharge again to verify their responses were consistent over time.

### Measures

Participants filled out measures needed to evaluate the outcomes in the larger study and to describe the sample. The screening survey included demographic and military characteristic questions, as well as the AUDIT to determine eligibility. All other items were included in the baseline survey. Participants also completed a follow-up survey that included items of gender, former branch of service, and rank at discharge. Only measures used for the current purposes are described here and other baseline and follow-up measures to achieve the larger study aims are discussed elsewhere [42].

#### Demographics and military characteristics

Participants filled out items regarding their age, gender, Hispanic ethnicity, race, marital status, education, income, current enrollment in college, current employment, number of children, and zip code. Participants also indicated their former branch of service, rank at discharge, pay grade at discharge, years of military service, number of years since discharge, months deployed as part of OEF; OIF; Operation New Dawn (OND); or other conflicts/operations, and their military occupational specialty; enlisted classification; or specialty code at discharge. Veterans also indicated how they learned about the study (i.e., from a Facebook ad, from a family member/friend) and on what device they complete the survey (i.e., mobile phone, tablet such as an iPad, personal computer or laptop, or public computer). Participants also completed an 11-item combat exposure scale developed from our prior work [9], with example items of “having a friend who was seriously wounded or killed” and “witnessing an accident resulting in serious injury or death.” Scores on the measure ranged from 0 to 11.

#### Treatment receipt

Participants indicated if they had ever since discharge used a VHA hospital or VHA community-based outpatient clinic for any services (i.e., mental health concerns, alcohol or other substance use concerns, physical exams, compensation and pension review). If so, they indicated whether they had used the service in the past year. Participants also reported how close they were to the nearest VHA hospital or community-based outpatient clinic using response options of “less than 5 miles away,” “5–10 miles away,” “11–20 miles away,” “21–30 miles away,” “more than 30 miles away,” and “I have no idea.”

#### Mental health and substance use

PTSD symptom severity was assessed with the 20-item PTSD Checklist for DSM-5 (PCL-5) [47]. The measure has been widely used for military and veteran populations and has adequate reliability and validity for young adult military samples [48]. A cut off score of 33 has been recommended as an optimal level to screen for possible PTSD diagnoses [49]. Depressive symptoms were assessed with the Patient Health Questionnaire 8-item (PHQ-8), which is a reliable and valid measure of depression used in research and practice for active duty military and veterans [50]. A cutoff score of 10 is recommended as reflecting a possible diagnosis of major depression. Sensitivity and specificity for the PCL-5 and PHQ-8 have been adequate in military samples in prior work [51–53]. The 10-item AUDIT assessed problem drinking in the past year, with a cutoff score of 8 indicating hazardous drinking [44]. Nicotine use (i.e., cigarettes, smokeless tobacco, e-cigarettes), marijuana use, and use of other drugs (e.g., cocaine, methamphetamine, opiates) were each assessed using single item measures with which participants indicated how many days in the past month they used each drug.

### Data analytic plan

Data analyses were primarily descriptive reports of frequencies, means, and standard deviations. We used chi-square tests to compare participants who completed the baseline survey with those who failed to complete the full baseline survey to determine whether demographic factors were associated with drop off prior to baseline survey completion. We also evaluated whether attrition at follow-up was predicted by any of the assessed baseline factors. We ran a logistic regression model predicting dropout from (1) demographic factors (gender, age, ethnicity, race, marital status, education, income, enrollment in college, currently working, manner where survey was completed [mobile phone/tablet vs computer], children [yes/no]), and a population code based on number of veterans per state [state identified from zipcode; states were categorized from 1 to 6 with 6 encompassing the states with the highest veteran population [54]], (2) military characteristics (branch of service, years of service, time since separation, combat severity, and number of months deployed as part of OIF, OEF, OND, or other conflicts), (3) treatment receipt factors (ever used a VHA, past year alcohol care at a VHA or elsewhere, past year substance care at a VHA or elsewhere, past year mental health care at a VHA or elsewhere, and distance from the nearest VHA), and (4) mental health problems and substance use (positive screens for the PCL, PHQ-8, and the AUDIT, nicotine use [including cigarettes, smokeless tobacco, and e-cigarettes], marijuana use, any other substance use).

## Results

### Findings from the Facebook advertisement strategies

The ads were funded on Facebook from 6/16/15 to 6/22/15 (7 days), at which point we had surpassed an initial target sample of 600 and turned funding off, but ads that contained comments or were tagged by users could still be viewed. We kept the survey live from 6/16/15 to 6/30/15 (15 days) to allow time for some individuals who started the survey to finish it, as well as to allow time for those who learned about the study from others to access and complete the survey (e.g., a spouse “tagged” a veteran in an ad). In total, we recruited a baseline sample of 793 young adult veterans that were verified through our verification procedures. We spent a total of $3,594.78 on Facebook ads, which translated to an advertising cost of $4.53 per obtained verified participant.

#### Ads targeting the general population of young veterans

These ads were shown to a total of 412,415 Facebook users, with a total number of 12,269 ad clicks. Nearly all (95%) of these clicks resulted from ads displayed on mobile phones. A number of Facebook users engaged with the ads, including 471 “likes,” 283 comments on the ads, and 391 ad shares (user either shared the ad on their personal page or they “tagged” another Facebook user in the ad to alert them of the study opportunity). Ninety-one percent of ad clicks came from men. The total cost of these ads was $2,824.61, with a cost per click of $0.23.

#### Ads targeting female veterans

These ads reached 50,986 Facebook users, with a total number of 996 clicks on the ads. Nearly all (94%) of these clicks resulted from ads displayed on mobile phones and all clicks were from women. Sixty-five users “liked” the ads, 51 left comments on the ads, and 110 shared the ad. The total cost of these ads was $378.84, with a cost per click of $0.38.

#### Ads targeting Air Force and Navy veterans

These ads reached 65,321 Facebook users, with a total number of 706 clicks on the ads. Again, nearly all (96%) of these clicks resulted from ads displayed on mobile phones. Forty two users “liked” the ads, while 27 left comments on the ads and 20 shared the ad. Ninety percent of ad clicks came from men. The total cost of these ads was $391.33, with a cost per click of $0.55.

### Recruitment flow and non-completion of the baseline survey

Table 1 details the participant flow into the study and reasons why individuals were exited from the survey or not retained for analyses. Of the 2,312 individuals that accessed the survey from Facebook, a total of 49.1% (N = 1,135) exited the survey immediately after clicking the ad or because they indicated that they declined to consent. Of the remaining 1,177 that went on to the screening survey for eligibility, 15% were screened out of the survey for multiple reasons. The main reasons were because they did not meet eligibility on AUDIT scores (7% of those screened), they indicated they were in the reserves/guard (4%) or still on active duty (3%), or were over the eligibility age of 34 (2%). Eleven participants were screened out of the study due to our mechanism verifying consistency between branch of service and rank at discharge (1%). At baseline, nine participants could not be determined as legitimate respondents. This was due to post-data collection checks that determined three participants completed the survey within an impossible time frame (less than eight minutes), one endorsed a job in the military that we could not verify against their rank/branch, and five participants (likely the same person) used the same email address to sign up for the study multiple times. For this latter issue, the potential participant must have created five separate Facebook accounts in order to gain access to the survey more than once, since one of the checks for limiting misrepresentation was to deny access to the survey website if a Facebook user had already completed the survey (or been screened out due to ineligibility).

**Table 1:** Participant flow.

	N
Clicked on Facebook advertisements to reach information page	13,971
2,312 clicked through information page to reach consent form	
Declined to consent	8
Consented and then exited the survey without filling out anything	1127
1,177 went on to screening	
*Screening items for verification*	
Active duty	34
Coast Guard	1
Reserves/Guard	44
AUDIT score less than 3 (women) or 4 (men)	84
Over age 34	20
Under age 18	0
Branch/rank did not match	11
1,002 went on to the baseline survey^1^	
*Post screening items for verification*	
Completed survey too quickly to be valid	3
Could not verify military occupational specialty, enlisted classification, or specialty code	1
Same email used to access survey multiple times	5
Did not finish	200
**Final baseline sample**	793
631 completed follow-up	
*Follow-up items for verification*	
Did not match on gender, branch, and rank between baseline and follow-up	4
Matched on gender, but not on branch and rank between baseline and follow-up	5
**Final follow-up sample**	622

^1^ 19 participants had more than one reason for exclusion so in the table they are counted either twice (17 had 2 reasons) or three times (2 had 3 reasons).

Of the 1002 that advanced to the baseline survey, 200 did not complete the baseline survey once they started it. There did not appear to be a section of the survey where participants were more likely to drop off. However, several demographic factors were associated with drop off from the baseline survey. In Table 2, we compare the demographic information of the baseline completers and participants who dropped off during the baseline survey. Men were more likely than women to begin the baseline survey and fail to complete it, *χ*^2^ (1) = 4.03, *p* = .045. Those with less education were also more likely to drop off during the baseline survey, *χ*^2^ (3) = 12.83, *p* = .005, and those who had ever used a VHA for services were more likely to drop off during baseline, *χ*^2^ (1) = 9.90, *p* = .002. In other words, women, those with more education, and those who did not use VHA services were more likely to complete the baseline survey and get randomized to the intervention study. In addition, drop off from the baseline survey appeared to also be a function of the device used to fill out the survey. Specifically, 89.5% of those that failed to complete the survey were on mobile devices or tablets, while 81.9% of the baseline survey completers took the survey using a mobile phone or tablet, *χ*^2^ (*N* = 992, *df* = 2) = 22.22, *p* < .001.

**Table 2:** Demographic Comparison of Baseline Survey Completers and Dropouts.

Variable	Sample that completed baseline *N* = 793	Sample that dropped off during baseline^1^
Age	*M* = 28.89 (*SD* = 3.38), Range = 19–34	*M* = 28.30 (*SD* = 3.82), Range 19–34
**Gender**^**2**^		
Male	83.0%	88.9%
Female	17.0%	11.1%
**Ethnicity**		
Hispanic/Latino(a)	10.6%	11.6%
Non-Hispanic/Latino(a)	89.4%	88.4%
**Race**		
White	84.5%	82.0%
Black/African American	4.2%	5.5%
Asian	1.0%	1.0%
Native Hawaiian or Other Pacific Islander	0.5%	0.5%
American Indian or Alaskan Native	2.4%	1.5%
More than one race	5.8%	4.0%
Other	1.6%	5.5%
**Education**^**3**^		
Less than grade 12 or GED completion	0.3%	0.0%
Completed Grade 12 or GED (High school graduate)	20.3%	25.6%
Completed some college or technical school	58.6%	64.3%
Completed college 4 years or more (College graduate)	20.8%	10.1%
**Branch of Service**		
Air Force	10.0%	7.5%
Army	59.5%	54.3%
Marine Corps	21.7%	26.6%
Navy	8.8%	11.6%
**Marital Status**		
Married	49.2%	45.2%
Separated	5.9%	5.6%
Divorced	13.6%	16.9%
Widowed	0.4%	0.0%
Never married	26.4%	23.7%
Engaged	4.5%	8.5%
**Income**		
$24,999 or less per year	28.9%	32.8%
$25,000 to $49,999 per year	38.8%	33.3%
$50,000 to $99,999 per year	25.4%	28.2%
$100,000 or more per year	6.9%	5.7%
**Enrollment in College**		
Currently enrolled in college	34.4%	32.8%
**Use of the VHA since discharge**^**4**^		
Ever used VHA for any reason	68.6%	79.7%

^1^N varied by item since not all 200 participants that failed to complete the baseline survey completed all items (e.g., only 91 participants were still active at the time they reached the VHA question); 199 completed age, ethnicity, race, and branch items; 177 completed marital status; 175 completed income and college enrollment items; 91 completed use of VA item.

^2^ Significant difference between completers and non-completers by gender.

^3^ Significant difference between completers and non-completers by education.

^4^ Significant difference between completers and non-completers by use of VHA since discharge.

### Description of the characteristics of the sample

#### Demographic characteristics

Table 2 contains the demographics for the sample. All participants were between ages 19 and 34 (mean = 28.9) and most were male (83.0%). Note that there were no 18 year olds recruited despite eligibility criteria specifying age of 18–34, likely due to the small number of individuals who enlist in the military at 18 and discharge early within that one year period. Eleven percent reported Hispanic ethnicity and most participants identified as White. About half of participants were married (49.2%). Sixty-two percent reported they had children and these parents reported a mean of 2.16 (*SD* = 1.52) children. Seventy-nine percent of participants reported completing at least some college and one-third (34.4%) were currently enrolled in college. Of those currently enrolled in college, 33.4% attended a community college, 26.7% attended a state university, 26% attended a private college or university, and 13.9% attended a technical college. Most participants reported full-time (55%) or part-time employment (11%), with 19% currently out of work and looking for a job and 15% out of work but not looking for a job.

#### Military characteristics

The veterans in our sample were spread across the four targeted branches of the military. Publically available data from the Department of Defense [55] indicated that our sample was most closely matched with the percentage of Marine Corps personnel that had separated from military service between 2010 and 2014 (21.7% in our sample, 20.1% in Department of Defense separated personnel population data), followed by separated Air Force personnel (10.0% in our sample, 15.4% in the separated personnel population). However, as in our prior work, we found the sample to overrepresent Army veterans (59.5% in our sample compared to 45.3% in the separated personnel population) and underrepresent Navy veterans (8.8% in our sample compared to 19.2% in the separated personnel population). As a reference, in our prior work, percentages by branches were as follows: 6.6% Air Force, 60.2% Army, 24.7% Marine Corps, and 8.5% Navy [32]. Thus, the targeted Air Force and Navy ads we developed helped to bolster recruited numbers of Air Force veterans. However, Navy veterans were still quite under-represented. It is important to interpret these findings with the caveat that our current sample was composed of drinkers (whereas our previous data did not screen for drinking) and available Department of Defense data could not be conditioned by age or drinking status. The Department of Defense data also defined “veteran” as including those in the reserve components of the United States Armed Forces and we excluded these individuals from our sample.

About half of the participants had separated from the military between 2001 and 2011 (49%), with the remaining 51% separating between 2012 and 2015. Participants reported a mean of 5.62 (*SD* = 2.80) years in the service. Most (82%) were deployed at some time during their service, with those who had deployed reporting a mean of 1.84 (*SD* = 1.11) deployments during their service. More than half of those deployed were part of OIF (61%) or OEF (56%), with 9% deployed as part of OND and 20% deployed as part of other conflicts/operations. Of participants who had deployed, 82% reported combat exposure, with a mean of 5.08 (*SD* = 2.91) on the 11-item combat exposure scale [9].

#### Survey modality

Most participants (95%) reported that they learned about the study directly from Facebook ads on a mobile phone (58%) or on a computer/laptop (37%). The remaining 5% were recruited by referrals from friends or family members either on Facebook itself (e.g., by sharing the link to the survey on Facebook, 3%) or by email (2%). Most participants completed the survey on a mobile phone (76%). Seventeen percent of participants completed the survey on a personal computer or laptop, 6% used a tablet (e.g., iPad), and 1% used a public computer (e.g., computer at a library). Two participants reported starting on a mobile phone and finishing on a personal computer.

#### Location

Participants came from 49 of 50 states in the United States, with at least one participant living in each state except South Dakota. The states most represented were Texas (N = 85), California (N = 55), Florida (N = 52), Arizona (N = 35), and Ohio (N = 35). Twenty or more participants lived in North Carolina, Illinois, New York, Pennsylvania, Georgia, Michigan, Missouri, South Carolina, and Colorado. Thirty four participants did not provide their zip code and thus state of residence could not be determined.

### Description of the behavioral health behaviors and service utilization of the sample

#### Overall use of the VHA

The majority of the veteran participants (69%) reported use of a VHA for some reason since separating from the military (see Table 3). When asked how far away the nearest VHA was from their homes, 11% reported it was within 5 miles, 25% reported it was between 5 and 10 miles away, 22% reported it was between 11 and 20 miles away, 14% reported it was between 21 and 30 miles away, and 22% reported it was over 30 miles away. Only 6% reported they did not know how far away the nearest VHA was.

**Table 3:** Service Utilization for the Full Sample and by Behavioral Health Concern.

	Past year mental health care	Past year alcohol use care	Past year substance use care	Ever sought care at VHA for anything since discharge
**Full sample**	317/793 (40.0%)	55/793 (6.9%)	49/793 (6.2%)	544/793 (68.6%)
**PTSD screen**	199/310 (64.2%)	39/310 (12.6%)	29/310 (9.3%)	252/310 (81.3%)
**Depression screen**	219/369 (59.3%)	42/369 (11.4%)	32/369 (8.7%)	284/369 (76.9%)
**Hazardous alcohol use screen**	242/564 (42.9%)	52/564 (9.2%)	41/564 (7.2%)	388/564 (68.7%)
**Past 30 day nicotine use**	202/482 (41.9%)	40/482 (8.2%)	37/482 (7.7%)	329/482 (68.3%)
**Past 30 day marijuana use**	77/178 (43.2%)	17/178 (9.5%)	27/178 (15.2%)	129/178 (72.5%)
**Past 30 day other drug use**^**1**^	53/108 (49.1%)	14/108 (13.0%)	23/108 (21.3%)	82/108 (75.9%)

^1^ “other drugs” not including nicotine, alcohol, or marijuana.

#### Use of the VHA and behavioral health care services

Table 3 contains past year service use at a VHA or non-VHA facility for mental health care, alcohol use care, and other substance use care for the sample as a whole and by number of participants screening positive for the behavioral health problems we assessed: depression, PTSD, and hazardous alcohol use.

For depression, participants reported a mean of 9.71 (*SD* = 7.12) on the PHQ-8. Nearly one half of participants (46.5%; *N* = 369) screened for possible major depression using a cutoff score of 10 on the measure [50]. Ninety seven participants (12.2%) screened for possible severe depression using a cutoff score of 20 or higher. Of those whom screened for possible major depression, 59.3% reported use of mental health care from a VHA or non-VHA facility in the past year. About three-quarters of these participants (76.9%) had ever sought care at the VHA for any reason.

For PTSD, participants reported a mean of 28.98 (*SD* = 22.86) on the PCL-5. Using a cutoff score of 33 to reflect individuals with a probable PTSD diagnosis [49], we found that 39.1% (*N* = 310) of the sample met this criterion. Of those whom screened for possible PTSD, 64.2% reported use of mental health care from a VHA or non-VHA facility in the past year. About 8 out of 10 participants with possible PTSD screens (81.3%) reported seeking VHA services for any reason since discharge from the military.

For hazardous alcohol use, participants reported a mean of 12.57 (*SD* = 7.06) on the AUDIT. Using a cutoff scale of 8 as a screener for hazardous use [44], 71.1% (N = 564) met this criterion, while 28.9% (N = 229) reported AUDIT scores between 3 or 4 (i.e., lowest value of participants per the study inclusion criteria) and 8. We also found that 29% (*N* = 230) met the criterion for hazardous drinking using 16 as indicative of problem drinking. Of those who screened positive for hazardous drinking, 9.2% reported receiving care for alcohol use from a VHA or non-VHA facility in the past year and 7.2% reported receipt of services for other substances. About two-thirds (68.7%) of those with hazardous drinking scores reported seeking VHA services for any reason since discharge from the military.

For cigarette/nicotine use, about half (44.5%) reported past month combustible cigarette use, with 20.4% reporting use of smokeless tobacco (e.g., dip, chew, snuff) and 18.3% reporting use of e-cigarettes. About one-fifth (22.4%) reported past month marijuana use. Concerning other drugs, prescription medicines to get “high” like Ritalin, Oxycontin, or Vicodin were the most used in the past month (6.7%), followed by amphetamine type stimulants (e.g., speed, diet pills, ecstasy) (3.2%), over the counter medicines to get “high” like cough/cold medicine or Dramamine (2.5%), cocaine (2.1%), and hallucinogens (1.4%). Less than 1% used other drugs such as synthetic marijuana, inhalants, methamphetamine, and heroin. Of those who used any nicotine in the past 30 days (i.e., cigarettes, chewing tobacco, e-cigarettes), 7.7% reported use of substance use care in the past year, and 68.3% reported ever using a VHA for any reason since discharge. Of those who used any marijuana in the past 30 days, 15.2% reported use of substance use care in the past year, and 72.5% reported ever using a VHA for any reason since discharge. Of those who used drugs besides nicotine, alcohol, and marijuana, 21.3% reported use of substance use care in the past year, and 75.9% reported ever using a VHA for any reason since discharge.

### Factors associated with dropout from the longitudinal study

At follow-up, 631 participants completed the survey. As noted in Table 1, nine participants could not be verified as the same participant from baseline based on inconsistent responses to demographic and military characteristic items. Thus, the final follow-up sample was composed of 622 participants. This represented 78.4% of the 793 participants who completed the baseline survey and 79.3% of the 784 participants that had consistent responses to demographic and military characteristic items across surveys.

We conducted logistic regression predicting dropout from the study (as defined as not completing the follow-up survey) where we entered in demographic factors, military characteristics, treatment seeking factors, and mental health problems and substance use, controlling for the intervention condition participants received (i.e., alcohol intervention vs. control). Findings from these analyses revealed that only education predicted dropout (*B* = 0.65, *SE* = 0.19, Wald *χ*^*2*^ (df = 1) = 11.11, *p* < .001), such that those with higher education were 1.9 times more likely to complete the follow-up survey. None of the military characteristics, treatment seeking factors, or mental health problems or substance use factors predicted dropout from the study at follow-up. Table 4 contains the model, including coefficients, standard errors, *p*-values, and odds ratios. As a sensitivity analysis to remove controlling for other factors, we similarly found that only education associated with dropout in bivariate models.

**Table 4:** Logistic Regression Model Predicting Dropout at Follow-Up.

	Coefficient	Standard error	Wald χ^2^	Odds ratio	95% confidence interval for odds ratio	
					Lower	Upper
Constant	-2.85	1.45	3.85	0.06		
Intervention condition^1^	0.00	0.23	0.00	1.00	0.64	1.56
**Demographics**						
Male	-0.12	0.39	0.10	0.88	0.42	1.88
Hispanic/Latino ethnicity	0.24	0.38	0.39	1.27	0.60	2.68
White race	0.26	0.34	0.58	1.30	0.66	2.54
Married	-0.06	0.25	0.05	0.95	0.58	1.53
Education	0.65	0.19	11.11	**1.91**	1.31	2.79
Income	0.11	0.10	1.14	1.12	0.91	1.37
Currently in college	0.22	0.26	0.73	1.25	0.75	2.09
Currently employed	-0.38	0.28	1.85	0.69	0.40	1.18
Number of children	-0.12	0.08	2.27	0.89	0.76	1.04
Completed survey on mobile or tablet	0.49	0.31	2.62	1.64	0.90	2.98
Veteran population within state	-0.03	0.09	0.08	0.98	0.82	1.16
**Military characteristics**						
Air Force veteran	0.12	0.47	0.07	1.13	0.45	2.84
Marine Corps veteran	0.11	0.28	0.16	1.12	0.65	1.92
Navy veteran	0.92	0.54	2.94	2.51	0.88	7.19
Years of service	0.05	0.05	0.75	1.05	0.94	1.16
Number of years since separation	0.00	0.05	0.01	1.00	0.92	1.10
Combat severity	0.00	0.05	0.00	1.00	0.91	1.10
Months deployed OEF	0.03	0.02	1.56	1.03	0.99	1.07
Months deployed OIF	0.00	0.02	0.02	1.00	0.97	1.04
Months deployed OND	0.06	0.07	0.82	1.06	0.93	1.21
Months deployed other conflict	-0.02	0.02	0.80	0.98	0.94	1.02
**Treatment seeking**						
Any use of VA since separation	-0.39	0.28	1.91	0.68	0.39	1.18
Mental health care in past year	0.44	0.28	2.58	1.56	0.91	2.68
Alcohol use care in past year	0.70	0.55	1.63	2.02	0.69	5.94
Non-alcohol substance use care in past year	-0.19	0.48	0.16	0.83	0.32	2.11
Distance to nearest VA	0.10	0.08	1.43	1.11	0.94	1.30
**Mental health and substance use**						
Hazardous alcohol use screen	-0.18	0.28	0.44	0.83	0.48	1.43
Possible major depression screen	-0.09	0.31	0.08	0.92	0.50	1.67
Possible PTSD screen	0.11	0.33	0.10	1.11	0.58	2.14
Tobacco use in past month	-0.07	0.24	0.07	0.94	0.58	1.51
Marijuana use in past month	-0.01	0.01	1.19	0.99	0.96	1.01
Other drug use in past month	-0.02	0.34	0.00	0.98	0.51	1.90

Significant odds ratio is bolded.

^1^ coded 1 for intervention, 0 for control.

## Discussion

In this study, we explored whether Facebook can be used to effectively recruit and retain participants in a brief alcohol intervention study tailored to young veterans at risk for heavy drinking and related consequences. Building on prior work recruiting young veterans for survey and intervention research [32, 34], we recruited a sample of heavy drinkers that were not specifically looking for care by using a series of ads not specifically geared toward those interested in treatment. The recruitment effort lasted about two weeks with a cost per obtained participant of $4.53. Participants all drank alcohol and were screened into the study based on relatively moderate criteria for heavy drinking. Thus, as a whole, the sample reported heavy drinking behavior that could warrant a brief intervention, but 71% reported hazardous alcohol use (AUDIT score of 8 or greater) that could clearly benefit from reduction efforts. Notably, only 9% of these hazardous drinkers reported receipt of alcohol use treatment in the past year. Thus, the Facebook recruitment effort was successful at locating those with hazardous use that had not sought recent treatment for potentially problematic use.

Recruitment via Facebook also enabled us to recruit a sample that was diverse with respect to demographic and mental health comorbidity. We obtained a sample of 17% women, which is slightly larger than the estimated 12% female OEF/OIF veteran prevalence reported by the Department of Veterans Affairs [56]. However, it should be noted that in order to do so, we needed to target ads directly toward female veterans. We similarly needed to go to extra effort to recruit veterans from the Air Force and Navy. Despite only mentioning “alcohol use” in ads, nearly half of participants screened positive for possible major depression and 39% screened positive for possible PTSD, which fits with documented high rates of heavy alcohol use and mental health comorbidity in the OEF/OIF population [3]. About 60% of participants reported nicotine use in the form of cigarettes, smokeless tobacco, or e-cigarettes, with one-fifth reporting marijuana use and about 14% reporting use of other drugs. Thus, rates of non-alcohol drug use were relatively high among this heavy drinking sample.

The Facebook recruitment was also helpful in attracting young veterans who were not seeking care for other behavioral health concerns beyond heavy alcohol use. For example, approximately 40% of those screening positive for PTSD or depression denied seeking past year mental health care and only approximately 11% of those using nicotine, marijuana, or other drugs reported past year substance use care. In addition, about 31% of the entire sample had never used VHA services since discharge. Most studies of OEF/OIF veterans recruit from within VHA clinics (e.g., flyers in primary care clinics, referrals from VHA providers) or use VHA medical records, and therefore, the non-VHA population of veterans remains largely understudied. Tapping into the population of non-VHA veterans through the use of alternate recruitment strategies such as Facebook is essential to better understand veteran behavioral health concerns. This study also demonstrates the website can be used to reach these non-VHA veterans and those not regularly seeking care at VHA or elsewhere and provide them with online services.

The process of the Facebook recruitment and retention of the sample can help to inform future work designed to reach and engage non-treatment seeking young veterans into online intervention studies. The response rate among those that clicked on ads was approximately 6% (i.e., 793 completed baseline of the 13,971 that clicked on ads). This low response rate is typical of Facebook studies [57] as many potential participants initially click on advertisements but most will drop off before completing screeners [32, 34, 58–60]. Though this response rate seems small, it includes large numbers of veterans with problematic drinking and high levels of comorbidity that seem unlikely otherwise to seek services. We do not have data on the reasons why many potential participants clicked ads but then failed to do anything once they reached our website. It is likely they realized at this point they were not eligible. Our ad targets focused on finding veterans and their family members, but ads were likely shown to many people with military interests would not necessarily be eligible (e.g., a supporter of a veteran services organization with no military affiliation). Still, factoring in that many people will click on ads and not complete a study is something researchers should consider when designing recruitment plans, especially since there is a cost associated with each ad click. It is also important to note that ads mentioned remuneration for study completion. Although study incentives are typically a focus of recruitment ads, it is unknown how Facebook can be used in efforts to engage young veterans in research and intervention programs without offering incentives.

Our analyses revealed that for this sample, failure to complete the baseline survey was a function of several factors; specifically, women, those with more education, those who did not use VHA services, and those completing the survey on laptop/desktop computers or tablets as opposed to mobile phones were less likely to drop off during the baseline survey. In this case, men, those with less education, those who used VHA services, and those on mobile phones were less likely to be randomized to receive the intervention study which they may have benefitted from. We need better ways to engage these individuals early on once they click on ads. A practical solution would be to make baseline surveys as short as possible to avoid drop off prior to receipt of interventions.

In the effort to inform research with participants recruited from Facebook, we attempted to learn what factors predicted dropout during the one-month longitudinal study. Approximately 80% of the sample was retained at follow-up. We were unable to find many differences between veterans who completed the follow-up and who did not. The *only* difference we found after exploring multiple demographic factors, military characteristics, treatment seeking factors, and mental health problems and substance use, was that veterans reporting more education were *less* likely to drop out of the study and not complete the follow-up survey. As those with less education were also more likely to not complete the baseline survey, this is a clear group that warrants more focused efforts to engage and retain during online alcohol intervention work.

## Conclusions

This descriptive feasibility study demonstrates that Facebook can be used to reach and retain a diverse sample of young adult veteran drinkers that could benefit from alcohol intervention efforts. Although we focused solely on Facebook recruitment, we suspect that use of social media, in general, is likely an effective strategy for reaching young veterans in need of alcohol interventions. Moreover, given that one-third of young adults additionally use Twitter and other short-status update sites such as Instagram and Snapchat [20], there are likely additional avenues by which young veterans can be reached outside of the VHA.

## Supporting information

S1 File Supplemental Dataset with Deidentified Data from Participants in this Study. [dataset PLOS ONE Facebook paper.xlsx].
(XLSX)
